# The International Pharmacy Game: A Comparison of Implementation in Seven Universities World-Wide

**DOI:** 10.3390/pharmacy9030125

**Published:** 2021-07-13

**Authors:** Tanja Fens, Denise L. Hope, Sarah Crawshaw, Eline Tommelein, Claudia Dantuma-Wering, Bertha Maria Verdel, Indrė Trečiokienė, Vibhu Solanki, Eugène P. van Puijenbroek, Katja Taxis

**Affiliations:** 1 Department of PharmacoTherapy, -Epidemiology and -Economics, Groningen Institute of Pharmacy, School of Science and Engineering, University of Groningen, Antonius Deusinglaan 1, 9713 AV Groningen, The Netherlands; c.m.dantuma-wering@rug.nl (C.D.-W.); e.vanpuijenbroek@lareb.nl (E.P.v.P.); k.taxis@rug.nl (K.T.); 2 Department of Health Sciences, University of Groningen, University Medical Center Groningen, Hanzeplein 1, 9713 GZ Groningen, The Netherlands; 3 School of Pharmacy and Medical Sciences, Griffith University, Gold Coast, QLD 4222, Australia; d.hope@griffith.edu.au; 4 Department of Pharmacy and Pharmacology, University of Bath, Claverton Down, Bath BA2 7AY, Somerset, UK; sc2715@bath.ac.uk; 5 Department of Pharmacy, Faculty of Medicine and Pharmacy, Vrije Universiteit Brussel, Laarbeeklaan 103, 1090 Jette, Belgium; eline.tommelein@vub.be; 6 Division of Pharmaco-Epidemiology & Clinical Pharmacology, Department of Pharmaceutical Sciences, Faculty of Science, Utrecht University, Universiteitsweg 99, 3584 CG Utrecht, The Netherlands; b.m.verdel@uu.nl; 7 Pharmacy Center, Institute of Biomedical Sciences, Faculty of Medicine, Vilnius University, M. K. Čiurlionio, Str. 21/27, LT-03101 Vilnius, Lithuania; indre.treciokiene@mf.vu.lt; 8 School of Pharmacy, University of Nottingham, University Park, Nottingham NG7 2RD, UK; Vibhu.Solanki@nottingham.ac.uk; 9 Netherlands Pharmacovigilance Centre Lareb, 5237 MH ’s-Hertogenbosch, The Netherlands

**Keywords:** pharmacy education, pharmacy curricula, collaboration, the pharmacy game, serious-game, simulation, medication safety

## Abstract

The utilization of serious games and simulations in health professional education has increased. The Pharmacy Game is one such concept that intersects gamification and simulation, in which pharmacy student teams competitively manage simulated pharmacies; a concept included in the pharmacy curricula of seven international universities. This study aimed to compare the implementation and conduct of the Pharmacy Game of participant universities and their students’ performance in the same educational task. Data were collected via a questionnaire completed by academic staff in April 2020, and the collation of results of the same patient case was conducted at each university (April 2020 to March 2021). The main results reflected differences in the game frequencies and the curricular approach (standalone or integrated course) and in the learning outcomes for the Pharmacy Game. Other differences were identified in the extent to which students of other professions were part of the game such as medical students or pharmacy assistants. Student case outcomes revealed similar strengths across the universities in patient communication and focus on safety, with variations identified as areas for improvement. Collation of the international utilization of the Pharmacy Game identified a broad spectrum of similar learning outcomes, inspiring a model of international *core* and *aspirational learning outcomes*. While the Pharmacy Game has been implemented with flexibility regarding the numbers of teams (4–10) and the duration of activity (12–36 days), all universities reported positive experiences and student outcomes, suggesting that the intervention represents a potential tool to deliver capstone learning experiences, promote interprofessional education, reinforce patient safety, and prepare pharmacy graduates for future practice.

## 1. Introduction

Serious games are increasingly employed in health professional education as engaging and motivational learning activities [1,2,3]. Serious games are strategies for education and training that apply game principles (e.g., goal, rules, challenges, and interaction) [4] for learning and skill acquisition. Under the umbrella of serious games are an array of potential of learning and teaching strategies, including gamified simulation, escape rooms, and computer games [2,5,6]. The benefits of simulation in health professional education are well recognized, allowing students to undertake repeated practice in a safe environment, without any risk of patient harm [7,8]. Simulation may be conducted virtually, face-to-face, or in a hybrid manner, as often occurred as a consequence of the COVID-19 pandemic [9]. Gamification in education means using some of the positive attributes of a game, such as story and competition [10]. Gamification of simulation enhances students’ motivation and engagement [11], and the motivational affordances include points, leaderboards, feedback, progress, and challenges [12]. Additionally, gamification may convey the consequences of participants’ actions and behaviors [13].

The Pharmacy Game is an innovative educational model, a serious game or gamified simulation, originally developed by the University of Groningen, Netherlands, under the title Groningen Institute Model for Management in Care Services (GIMMICS^®^) [5,14]. In the Pharmacy Game, student teams competitively manage their own simulated pharmacies in a fully immersive, in-person simulation. It was designed to deliver capstone learning experiences intended to integrate and synthesize all prior learning, providing a culminating experience toward the end of the student’s study [15,16]. This aligns with the concepts of a spiral curriculum in which iterative revisitation of topics occurs throughout a degree program [17]. Informed by self-determination theory [18,19], the game includes deliberate and planned learning outcomes, to enhance the competence, confidence, collaboration, and preparedness of pharmacy graduates [5,14,20]. A unique attribute of the Pharmacy Game is that students are given the autonomy to put themselves in the pharmacist’s role, to make clinical and professional decisions safely, in an authentic environment that closely simulates their future real-world practice [21]. This authenticity supports the development of students’ professionalism and professional identity [22,23]. During the Pharmacy Game, student teams perform tasks common to community pharmacists, such as developing pharmacy business plans, checking prescriptions, dispensing medications, counselling patients on medication utilization and safety, as well as collaborating with other health care professionals. The simulated pharmacies start the game with an allocation of “patients,” and all tasks scored will either earn or lose patients for their team, dependent on the students’ performances. Poor performances or illegal behavior would result in patients being lost, whereas professional behavior and positive student performances result in patients gained. All activities are marked continuously with scores published through the web tool of the pharmacy game. The variety of learning tasks able to be presented during the Pharmacy Game reinforces and assesses students’ knowledge, skills, and attitudes, addressing the three domains of learning identified in Bloom’s taxonomy: cognitive, psychomotor, and affective learning [24,25]. Students receive regular indirect and direct feedback on their performances, individually or as a pharmacy team. Students compete to be the highest-scoring pharmacy as the pharmacy team with the most patients will be pronounced the winner of the game.

To date, seven universities from five different countries have implemented the Pharmacy Game into their curricula. The implementation and utilization of this educational model has not been studied across the universities. The universities utilizing the Pharmacy Game are diverse and spread from Europe and the United Kingdom to Australia. The various Pharmacy Game participant universities have evaluated the outcomes of their respective serious games, using various approaches and an array of lenses, including but not limited to critical self-reflection [26]. Each university has made ongoing iterative improvements to their game based on their own observations and reflections. Additional sources of feedback include student evaluation and graduate feedback, peer observation, and feedback both from the pharmacy profession and interprofessional colleagues. The University of Groningen has published descriptive studies on the Pharmacy Game, GIMMICS^®^ [5,14,27]; Utrecht University has published research related to their Pharmacy Game [20,21,23]; and the Vrije Universiteit Brussel published on the application of their Pharmacy Game, GIMMICS^®^, in medical students [28]. Griffith University recently published on students’ experiential learning in their Pharmacy Game, PharmG [29]. Additionally, several of the Pharmacy Game universities have presented student outcomes and preliminary research results at international conferences, including the University of Groningen [30,31], Utrecht University [32,33], University of Nottingham [34,35,36,37], and Griffith University [38,39,40,41,42]. All have adopted and adapted the Pharmacy Game to their own country’s practice environment and their university’s educational context and curricular needs, applying their own rules, requirements, and nomenclature to the Pharmacy Game. Research to collate the similarities and differences in implementation and utilization has not yet been conducted. Such findings inform researchers and educators about the possibilities of integrating a pharmacy simulation game in their curriculum. Thereby, the research supports the advancement of the education and training of pharmacy students. This study aimed, firstly, to compare the implementation and conduct of the Pharmacy Game of the seven participant universities, in particular focusing on the game utilization, frequencies, and learning outcomes pursued at each university. Secondly, it aimed to explore the assessment approaches and student performance across the different universities when undertaking the same educational task during the respective iterations of the Pharmacy Game.

## 2. Materials and Methods

This study used a mixed methods approach. Firstly, a survey method was applied. A structured questionnaire was developed to enable uniform questions to be asked of a variety of participants and to compare and contrast the answers [43]. Secondly, an educational task was developed, which was included in each university’s game, and a comparison was made of the assessment approaches and student performance in the different educational settings across the seven universities. 

Descriptions and reporting on the Pharmacy Game have been guided by the Standards for QUality Improvement Reporting Excellence in Education (SQUIRE-EDU) guidelines [44]. These guidelines were deemed the most suitable guidelines as they are specifically intended to improve the reporting of interventions in health care education. The three key components emphasized in SQUIRE-EDU are a description of the local educational gap; impacts of the intervention on patients, families, communities, and the health care system; and identification of iterative improvement and modification of interventions [44].

### 2.1. Study Design and Methodologies for Implementation and Conduct of the Pharmacy Game

#### Questionnaire Design, Distribution, and Data Analyses

**Figure 1:**
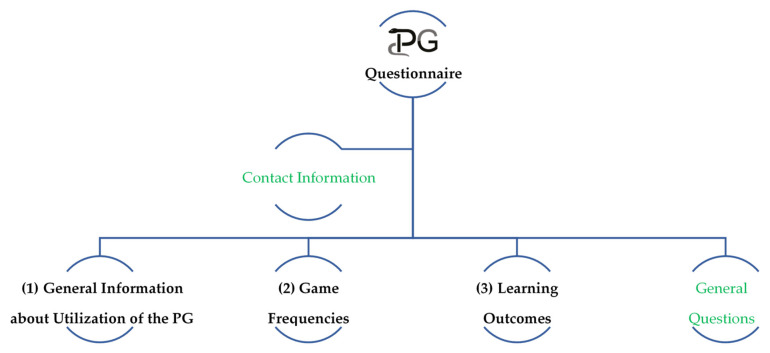
Questionnaire structure; PG—Pharmacy Game.

The research team of the University of Groningen (T.F., K.T., and C.D.W.) drafted the first version of the questionnaire, using both closed- and open-ended questions, categorized in several groups of questions (3 main categories) as presented in Figure 1. Then, the questionnaire was tested and distributed to all universities. The questionnaire followed a structure starting with opening questions about contact details of the participants and their game, categorized as contact information. The next three categories sought details about (1) how the game was introduced and used in the pharmacy curricula; (2) the frequencies of conducting the game; and (3) the main learning outcomes and assessment methods. The questionnaire concluded with an open response opinion question, providing space for additional comments. The full questionnaire is available in a Supplementary File (Table S1). 

The questionnaire was created in Qualtrics (Qualtrics XM), pre-tested by six experts (three pharmacists: T.F., K.T., and C.D.W.; two pharmacy assistants: E.Š.A. and C.d.V.V; and one information technology engineer: J.P.), and chosen based on previous experience with questionnaire-related studies. Minor content and design adjustments were made based on pretesting. The questionnaire link was electronically distributed to the Pharmacy Game participant universities by email, and email reminders were sent to encourage completion within the predefined timeframe. The questionnaire automatically auto-saved responses and could be completed over multiple attempts. By design, respondents were identifiable (as blinding does not add any additional value of the study), and it was estimated that the questionnaire took around ten minutes to complete. Data collection was conducted in April 2020. 

The questionnaire was distributed to all seven universities that had implemented the Pharmacy Game, namely, the University of Groningen [5,14,27] and Utrecht University [20,21,23], Netherlands; Vrije Universiteit Brussel [28], Belgium; University of Nottingham and University of Bath, England; Vilnius University, Lithuania; and Griffith University, Australia [29]. One representative from each university was the contact person completing the questionnaire. Therefore, ethical clearance was considered unnecessary, and a corresponding statement is provided in a Supplementary File (see Supplementary Materials).

Upon questionnaire completion, several follow-up consortium meetings were organized, during which the content of the questionnaire was clarified (in case of unclear or vague answers) and responses confirmed. Participants provided some additional information at this time to complement the questionnaire, e.g., year of study in students’ program, prerequisite student learning, simulated patient cases, and timing of common case. Results were extracted and presented descriptively [45] per the investigated category, as presented in Figure 1. Data were analyzed in two phases. First, two researchers (T.F. and D.L.H) structured the data presentation to best reflect the research goals. Then, six more researchers (S.C., E.T., C.D.W., B.M.V., I.T., and V.S.) representing each university contributed to the final data interpretation, thereby adding to the validity of the research [46]. Content analysis was used to analyze the qualitatively presented data, and summative content analysis was used to present quantitative outcomes such as students per game, students per team, staff per game, or annual interactions [47]. Finally, all data on the learning outcomes were reviewed to identify a common suite of learning outcomes. These learning outcomes were then categorized as core to the Pharmacy Game, defined as learning outcomes used by all universities, and aspirational outcomes, defined as learning outcomes applied by some universities. The learning outcomes were then mapped in a structure based on Bloom’s Taxonomy of Educational Objectives, as it is a widely accepted educational taxonomy and represents the broad learning opportunities in the Pharmacy Game [25]. 

### 2.2. Study Design and Methodologies for Assessment Approaches and Student Performance in the Different Universities

#### Patient Case Selection, Content, and Data Analyses 

During a consortium meeting of participant university partners, it was agreed that the same patient case would be conducted at all seven universities during one of their upcoming Pharmacy Game iterations (between March 2020 and March 2021). A number of cases were initially proposed and discussed, with advantages and disadvantages of each identified. It became apparent that not all medications under discussion were universally available, so the focus was then on identifying a case that would not be complicated by lack of universal availability. The issue of cultural diversity and comprehensive inclusion of different patients’ backgrounds was considered. Finally, all agreed to adopt a dermatological patient case. The research team of the University of Bath provided this case (see Supplemental Material) to be distributed to all participant universities. 

The patient case involved a simulated patient presenting to the simulated pharmacies as a parent whose child had uncomplicated eczema and darker skin. While personal details of the case may be amended, it was anticipated that the simulated patient or actor in each of the universities would present the case consistently, while visiting all the pharmacies managed by the students. As with all simulated patient cases, it was expected that information would only be revealed to the student-pharmacist in response to well-targeted and contextualized questions. Scenarios were designed to take no longer than ten minutes, and the simulated patients were expected to keep to this time. The simulated patient briefing notes for the eczema case are available in the Supplemental Material (Table S2).

After conducting the patient case, each university sent the assessment forms, reflecting the students’ performances to the two researchers (T.F. and D.L.H.) who initially extracted and analyzed these data. Then, six more researchers (S.C., E.T., C.D.W., B.M.V., I.T., and V.S.) contributed to the final data interpretation ensuring better validity of the research [46]. The outcomes of each patient case assessment were compared to determine whether any university’s approach might inform others in the consortium and where opportunities might exist for improvement. 

## 3. Results

### 3.1. Implementation and Conduct of the Pharmacy Game

All seven (100%) of the Pharmacy Game participant universities completed the questionnaire on their specific approaches to the game. The results for the majority of questions are summarized in Table 1 and explained in the forthcoming Section 3.1.1, Section 3.1.2 and Section 3.1.3. The general questions, including free text responses on additional experiences with the Pharmacy Game, have been collated in the Appendix A (Table A1).

#### 3.1.1. General Information about Utilization of the Pharmacy Game 

While most universities offered the Pharmacy Game as a standalone course, some delivered it as part of an integrated larger course or courses (see Table 1). The Pharmacy Game was considered mandatory in all locations.

Five of the seven universities reported European Credits (ECs) associated with their Pharmacy Game. The system was developed to facilitate student mobility and academic recognition [48]. It was not reported in association with the Pharmacy Game by the University of Nottingham or Griffith University.

The additional information provided by participants during follow-up meetings informed us that all universities insert the Pharmacy Game into their curriculum for senior students (4th, 5th, or 6th year of the program). The expectations of senior students at all universities is that students carry forward all prerequisite learning, including knowledge, skills, and capabilities, that are delivered earlier in the respective degrees. Universities reported a range of frequencies for delivering simulated patient cases, usually from one to six different cases with actors each day.

#### 3.1.2. Game Frequencies and Game Management Staff

There was much variation in the annual iterations of the Pharmacy Game delivered (from one to four times per academic year); the duration at each site (from 12 to 36 days); the number of students that participated in each Pharmacy Game iteration (from 24 to 60 students); and the number of students manning each pharmacy (from 5 to 12 students). However, the number of teaching staff manning the Pharmacy Game was consistently small (between two and five staff per location). Note, the full-time staff member equivalence reported in Table 1 related to those directly involved in the game management. Staff generally included academic staff, pharmacists, and pharmacy assistants with Groningen and Vilnius Universities specifically reporting on the additional use of physicians. In addition to pharmacists and pharmacy academics, most universities reported on the involvement of technical staff, research students, or others in support of their Pharmacy Game. Information technology (IT) or technical services specialists were reported by six of the seven universities, being used to build up the pharmacies’ digital needs, to set up the patient and prescription databases, and to prepare physical resources for each Pharmacy Game iteration. The University of Nottingham did not specifically report on local IT support but that they utilized the support inherently offered centrally from University of Groningen to all Pharmacy Game consortium member universities.

#### 3.1.3. Learning Outcomes and Assessments

The learning outcomes identified by each Pharmacy Game participant university have many commonalities but were also presented quite differently. At a minimum, universities included outcomes on communication, teamwork, and collaboration. Most universities also reported outcomes focused on the acquisition of knowledge and competence. Some of the learning outcomes were aspirational. For example, the University of Groningen used the term “pharmaceutical expertise,” whereas the University of Nottingham and Griffith University used the terms “advanced” for the higher-order expectations of student achievement. Despite the commonalities, the number and detail in each university’s learning outcomes varied. Griffith University and the University of Nottingham were the only institutions to report learning outcomes associated with the expected level of application from Miller’s (1990) pyramid of clinical assessment [49]. The reported learning outcomes are summarized for each university in Table S3.

**Figure 2:**
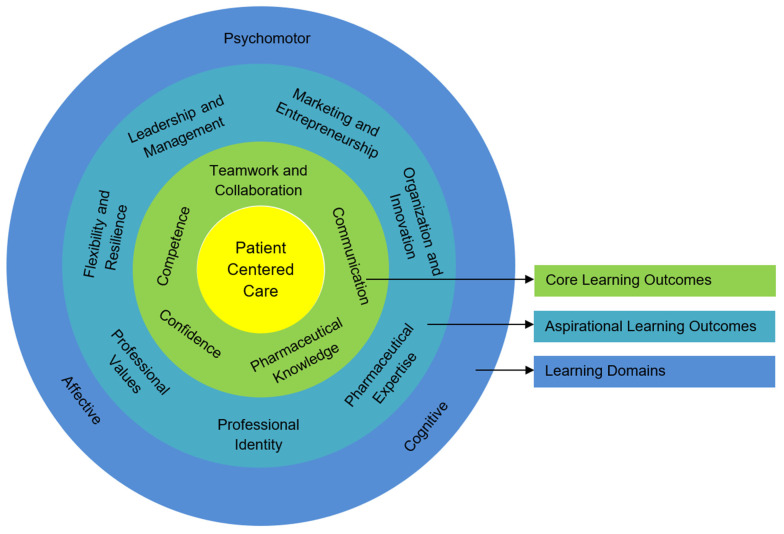
Proposed universal learning outcomes for the Pharmacy Game.

As the Pharmacy Game enables learning and teaching in the psychomotor, cognitive, and affective domains of Bloom’s taxonomy [25], it was used as the base structure for summarizing and aligning the reported learning outcomes (Figure 2). A common suite of learning outcomes has been recognized, with both core and aspirational outcomes identified, in a structure overlaid against Bloom’s [16] domains. 

One contrast in the Pharmacy Game delivery is the issue of individual student assessment. While all universities reported that team assessment was conducted, only one university did not conduct individual student assessment. In this area there was further variation as some universities reported separate individual student scores during the Pharmacy Game (Groningen, Utrecht, Nottingham, and Bath) for communication, pharmaceutical expertise, handling medication monitoring signals (only Utrecht), or reflection. Griffith University reported only team scores during the conduct of the Pharmacy Game and provided for students’ peer scaling to generate concluding individual student scores. 

Most universities provided regular ongoing marking of all tasks and activities within their respective Pharmacy Game offerings. Some universities (Groningen, Utrecht, and Brussels) attached an overall pass or fail requirement for students, whereas for others the results of the game provided a graded contribution.

While the gamified approach to the Pharmacy Game is replicated at each participant university, the approach to scoring and the number of “patients” awarded for each assessed activity within the Pharmacy Game varies. The starting scores, mean winning scores, and individual marks granted for sub-tasks are summarized in Table S4 (see Supplementary File). Details regarding the scoring system were provided in previous publication [5].

#### 3.1.4. Additional Aspects Identified within the Pharmacy Game

While all universities reported that they used the Pharmacy Game specifically to train pharmacy students, several universities extended the scope of their game to incorporate interprofessional collaborations with other health care professionals in training or practice (see Table 2). 

In Groningen, collaborations were undertaken with pharmacy assistants in training (who experienced consultations in the simulated pharmacy, as well as pharmacy recruitment interviews) and medical students (who simulated the role of general practitioners that consulted with the student-pharmacists to optimize patient treatments and medication utilization). In Utrecht, teacher-pharmacists and general medical practitioners provided joint training in interprofessional collaboration. Practicing pharmacists were involved either as a staff member or as simulated patients. In Belgium, simulated pharmacies were visited by practicing pharmacists to conduct cases. Additionally, all pharmacies were contacted by practicing physicians, midwifes, and nurses to inquire about medication-related issues. The University of Nottingham also used training pharmacists and simulated patients as actors and assessors. The final-year students worked on various projects with other final-year students from schools within the university. Business school postgraduates assessed and provided feedback on pharmacy business plans, and dietetic students collaborated on a complex patient case. Pre-pandemic discussions were at an advanced stage regarding future collaborations with medical, nursing, and midwifery schools pre-pandemic. At Griffith University, practicing pharmacists participated in the Pharmacy Game as assessors or simulated patients, and School of Medicine academic staff were involved in case delivery, debriefing, and feedback. At the University of Bath, practicing pharmacists from within the department and externally participated in the game as assessors or simulated patients. At Vilnius University, a lecturer-general-practitioner participated in the Pharmacy Game as the assessor of the clinical pharmacist’s cases, and medical students contributed as simulated patients. The incorporation of elements of interprofessional collaboration in each pharmacy game was mainly driven by the appeal of the activities incorporating collaborative student learning. Furthermore, two universities introduced this educational tool to additionally educate medical students [28]. The Vrije Universiteit Brussel implemented GIMMICS to their medical students as an addition to internships in family practice. They reported successful outcomes for medical students in the areas of communication and collaboration [28]. 

### 3.2. Student Performance on the Patient Case in the Different Universities

Six of the seven participant universities implemented the mutually agreed upon patient case in one of their Pharmacy Games in the period between March 2020 and March 2021. The student outcomes are presented in Table 3, while the potential case management options and case assessment variables are provided in a Supplementary File (Table S5).

Assessment forms reflected the potential pharmacological and non-pharmacological case management options and the varying aspects of communication. Most universities used their existing Pharmacy Game marking rubrics for the case. The pharmacological recommendations naturally differed by region based on the availability of appropriate medicines for pharmacist provision. In England, Belgium, Australia, and Lithuania, some mild to moderately potent topical corticosteroids were available for pharmacists to recommend, such as hydrocortisone (Australia, Belgium, England, and Lithuania), triamcinolone acetonide (Belgium), and clobetasone (England, Australia, and Lithuania). The participant universities were consistent regarding expectations of students’ non-pharmacological recommendations for the dermatological case, which included advice such as trigger avoidance. Aspects of communication that were assessed were also fairly consistent, with most universities expecting students to provide suitable counselling on the safe and appropriate use of the recommended medication, including treatment expectations, potential adverse events, and referral advice to a medical professional in the event of a worsening condition or adverse events. These aspects of assessment reflect the culture of patient safety being encouraged and reinforced in the Pharmacy Game. Overall, the student outcomes demonstrated that the best reported aspect was the patient communication, whereas areas for improvement mostly related to non-pharmacological advice and trigger identification. Some students did not sufficiently communicate counselling regarding medication safety, in aspects such as potential adverse effects, which was considered especially important when the recommenced treatment was over the counter (OTC). Patients are solely reliant on the pharmacist’s advice for following OTC medicines provision. Students also struggled to make the appropriate diagnosis, as the patient had darker skin. The differential diagnosis was better reflected at the universities that conducted the case in the later stages of the game. 

## 4. Discussion

### 4.1. Main Findings

The key findings of this study highlight the various ways that universities have adapted the Pharmacy Game educational concept to their own national and curricular contexts. Similarities were identified in all universities’ learning outcomes as each intended to train pharmacy students with the requisite knowledge, skills, and behaviors to prepare their graduates for the real-world environment of a range of pharmacy practice settings, including community and clinical pharmacy. The importance of such training was emphasized by the mandatory nature of the educational concept in all participant universities. Differences between universities were identified in the approach to introduction into the curricula (standalone versus integration into an existing course), the number of annual iterations, game duration, the number of students and staff members involved, as well as differences in the assessment methods (team versus individual assessments). The universities that participated in the eczema patient case during their Pharmacy Game reported good communication skills and pharmacological treatment recommendations by their students. 

### 4.2. Interpretation

#### 4.2.1. Implementation and Conduct of the Pharmacy Game

In contemporary education, serious games have taken a more important role in shaping pharmacy curricula, and they are being incorporated as well-accepted and evidence-based teaching methods [50]. A review study [51] exploring lower fidelity educational games implemented into pharmacy curricula showed that such tools are often implemented as a part of existing courses, including pharmacotherapy [52,53], clinical pharmacy [54], pharmacy practice [55,56], or professional communication [57,58]. Higher fidelity games involve students engaged in clinical or practice-based scenarios that replicate real-world practice [59]. In the context of this study, the immersive simulation of the Pharmacy Game is usually conducted as a standalone course but is integrated into a pharmacy practice course at the Vrije Universiteit Brussel and is imbedded across two courses at Griffith University, namely, pharmacy practice and pharmacotherapeutics. The unique design and flexibility of the Pharmacy Game enables it to be offered in different curricular modes, allowing diverse aspects of the pharmaceutical practice to be combined into the required educational tool.

The variations in the team numbers and game duration identified with this research may impact the staffing requirements and budgets of participant universities. Moreover, the students’ experiences, irrespective of the game duration (in our cases, between 12 and 36 days) depended on the learning goals the universities wanted to achieve as well as the curricula capacity of the pharmacy program at each university. This indicates the flexibility of this educational concept in providing students the authentic experience of stepping into the pharmacist’s role.

While some serious games and patient simulation methods used in healthcare professional education often focus on a singular area of training, such as communication [57,58] or pharmacotherapy [60] or interprofessionalism [61], the extended duration and immersive nature of the Pharmacy Game allows for a broad spectrum of learning outcomes to be achieved. The core intended learning outcomes of competence, confidence, communication, pharmaceutical knowledge, and teamwork and collaboration help to prepare the students to become work-ready professionals who will apply legal, ethical, and clinical reasoning in their future practice, with a focus on patient safety and optimal health outcomes. As all learning outcomes (Figure 2) revolve around patient-centered care, this signifies that the patient and their positive outcomes should be central to all learning [62]. As such, core learning outcomes should be evident in all iterative deliveries of an educational approach such as the Pharmacy Game. The emphasis of the learning outcomes can aim towards the aspirational, which may suit the context of some locations, and potentially increase the work-readiness of pharmacy graduates. The aspirational learning outcomes identified include skills and knowledge development that encourages pharmaceutical expertise; organizational skills; leadership, management, and skills in negotiation; innovation; marketing and entrepreneurship; or competence and confidence that facilitates professional values and identity [22]. Attributes such as flexibility and resilience, while desirable, might challenge educators to design and deliver components of their simulation to meet these goals, yet these future-facing outcomes are attributes expected in the future pharmacist workforce [63]. The proposed model of international learning outcomes may provide a base from which other Pharmacy Game participant universities might develop their constructive alignment and enhance the transparency and clarity of student expectations.

While the pharmacist’s role is shifting towards patient care [62], best practices are achieved when all health care professionals are involved in interprofessional collaboration [64]. Teaching students in interprofessional collaboration is still very fragmented and often absent [65]. Practicing interprofessional education brings students from different health professional perspectives into a mutual learning environment to complement each other towards optimal patient care [66]. It is well recognized that simulation affords opportunities to enhance interprofessional collaboration and communication skills [67], and while the main focus of the Pharmacy Game participant universities is to train pharmacy students, elements of interprofessional education were identified within this game concept. Introducing interprofessional collaboration in educational settings may be challenging and time-consuming as different educational groups should be engaged, which may be coordinated and implemented in practice. However, the experiences reported by the seven universities indicated that their colleagues from allied healthcare professions were enthusiastic to participate in such a valuable learning experience, as gamifying a simulation adds fun and is engaging and motivating. Herewith, a great opportunity opens for enhancement of the Pharmacy Game outcomes by further expansion into interprofessional education. Simulation of true multidisciplinary collaboration has the potential to provide authentic student experiences that they can carry into their future practice [68,69,70,71]. 

#### 4.2.2. Student Performances on the Patient Case in the Different Universities

It was a unique experience to have the students from all around the world conduct the same eczema patient case. This activity revealed differences in available treatment recommendations across the countries, the variations in the assessment methods, areas where students showed good knowledge and competence, as well as areas for improvements. It clearly showed that recommended treatment options varied across the participant countries, as different OTC and prescription medications were available and recommended for the condition in the respective locations. Moreover, assessment forms, as well as the communication of the safety issues with the patients, slightly differed. Aspects of patient safety, such as counselling on a medicine’s potential adverse events and how to manage them, were not always discussed with the simulated patient during the eczema patient case. This might have been a result of the less severe condition of the patient, and the possibilities for OTC treatment. However, addressing the potential adverse events of a medicine was an expectation in the assessments across all the universities, implying that more attention might need to be applied on this aspect of counselling in future cases of a similar nature. It has been suggested that the medication safety is rarely a focus of the serious games [72]. Some evidence from a study comparing an educational game activity with an e-module on increasing the medication safety knowledge showed that both approaches reflected equivalent learning among the students [73]. Unlike the previous evidence, another study showed that integration of medication safety within educational tools for experiential teaching contributes to improved patient care [74]. Similarly, the Pharmacy Game involves various elements on medication safety. Nevertheless, educational games are useful tools for improving students’ perceptiveness, allowing for the practice of communication skills such as being non-judgmental and showing empathy when interacting with simulated patients [75]. As the eczema case intentionally included a patient with darker skin, it allowed for testing of some of the previously mentioned perceptions of students. Finally, there was a connection identified between the students’ performances and the timing of when the case was conducted. When conducted later in the game, students displayed greater confidence to respond to the challenges of the case, which was reflected in better outcomes, especially in the aspect of appropriate differential diagnosis.

### 4.3. Strengths and Limitations

Serious games are often used as innovative approaches in higher education, mostly focusing on particular learning goals. This study demonstrated that the educational concept of the Pharmacy Game allows for teaching of a wide range of skills and behaviors, enabling attainment of a range of learning outcomes. The concept was shown to be a useful platform for practicing interprofessional education. For example, students from Griffith University positively reflected upon the interprofessional learning opportunities from their Pharmacy Game and welcomed the opportunity to simulate professional exchanges with medical professionals [29]. Moreover, to the best of our knowledge, it is the only pharmacist educational tool based on the convergence of simulation and gaming, used by multiple universities.

There were several recognized limitations to this study. Firstly, a questionnaire was used to gather basic data with answers then clarified in additional discussion with respondents. There may be more subtle differences and similarities in the implementation of the Pharmacy Game that may have been revealed using other methods. The authors believe that the data presented in this article provide a comprehensive overview and insight into the most important differences and similarities across the participant universities. Secondly, a single educational task was used to similarly assess students of all participant universities. Perhaps a variety of different tasks could be delivered to collect more data and gain additional deeper insight into differences and similarities across the universities. Further work may address this aspect. Moreover, the standardization of the patient case assessment form was complicated by the jurisdictional variation in pharmacological treatment options available for the pharmacist to recommend without prescription. It is therefore recommended that future patient cases conducted across institutions use the same standardized marking rubric, to better facilitate the head-to-head comparison of student outcomes and performances. Note, such standardization should not interfere with the already established assessments that each university created and aligned with their national and educational contexts and requirements. Conducting the patient case across the universities was, however, a useful activity to emphasize the differences in assessment models across the universities.

### 4.4. Research Implications

Additional research on student outcomes in the Pharmacy Game is needed to demonstrate students’ enhancement of self-assessed professional competencies and improved affective learning, as a consequence of participation in the Pharmacy Game. The outcomes for improved affective learning are particularly notable as it is the domain of learning that is arguably the most difficult to teach and is essential to the development of professional values and identity. In the future, it is essential to present more research on student and graduate outcomes as a consequence of participation in the Pharmacy Game. Future research could potentially explore students’ physiological measures of, and their self-reported, stress during this type of activity compared to more traditional learning and teaching activities. Additionally, it may be valuable to research methods to assess student engagement and motivation during the Pharmacy Game, as well as potential tools to achieve those research aims.

## 5. Conclusions

The implementation and conduct of the Pharmacy Game educational concept within the pharmacy curricula of seven universities was revealed as a mandatory activity in all universities and was predominantly incorporated as standalone course (five universities), as opposed to an integrated one (two universities). Students were assessed as teams (seven universities) and additionally as individuals (six universities). Evident differences were recognized in game frequencies and staffing with variations in annual iterations (1–4), game duration (12–36 days), students per game (24–60), pharmacy teams (5–12), and game management staff (2–5). Such variations were a consequence of the curricular capability of the pharmacy program and the learning goals of each university. A model of international learning outcomes has been proposed including *core learning outcomes* (communication, teamwork and collaboration, competence, confidence, and pharmaceutical knowledge) and *aspirational learning outcomes* (professional identity, pharmaceutical expertise, organization and innovation, marketing and entrepreneurship, leadership and management, flexibility and resilience, and professional values) in three learning domains (cognitive, affective, and psychomotor). Moreover, the educational concept of the Pharmacy Game based on the experiences of seven international universities represents a potentially useful tool for delivering a capstone learning experience, promoting interprofessional education, practicing medication safety, and preparing pharmacy graduates for future practice.

## Figures and Tables

**Table 1:** The Pharmacy Game characteristics across the participant universities.

University	University of Groningen	Utrecht University	Vrije Universiteit Brussel	University of Nottingham	Griffith University	University of Bath	Vilnius University
**Country**	The Netherlands	The Netherlands	Belgium	England	Australia	England	Lithuania
Year *	2000	2004	2008	2015	2016	2018	2019
**Name of the game**	GIMMICS	GIMMICS/ PharmacyGame	GIMMICS	GPPG	PharmG	GPMS	GIMMICS Vilnius
Case Management System (CAMS) **	Y	A	A	Y	A	N	N
Actor Registration System (ARS) **	Y	A	A	A	A	N	N
Prescription Generator **	Y	A	N	A	A	A	A
**Mandatory Activity**	Y	Y	Y	Y	Y	Y	Y
**Type of Course**	S	S	I	S	I	S	S
**Associated ECs**	Y	Y	Y	N	N	Y	Y
**Number of ECs**	8	7.5	30	-	-	6	5
**Annual Iterations**	3	4	1	4	1	3–4	1
**Game Days per Game**	25	20–25	20	36	15	12	12
**Students per Game**	20–36	30–49	60	60	50	30	24–26
**Students per Team**	5–6	5–7	12	6	5–8	5	5–6
**Staff per Game (FTE)**	2–3	2-4	4	2	5	2	2
**Team Assessment**	Y	Y	Y	Y	Y	Y	Y
**Individual Assessment**	Y	Y	Y	Y	Y	Y	N

* Year of implementation of the Pharmacy Game; ** Case Management System, Actor Registration System, and Prescription Generator are software tools for facilitating the game management. Their functionalities and definitions are given in previous publication [5]. GIMMICS=Groningen Institute Model for Management in Care Services; GPPG= Pharmacy Leadership and Management; PharmG = Groningen, Griffith, Gold Coast Game; GPMS= Groningen Pharmacy Management Simulation; Y = yes, N = no, A = alternative ways to manage; S= standalone, I = integrated; ECs = European Credits; FTE = full-time equivalent.

**Table 2:** Aspects of inter-/intra-professional collaboration in the Pharmacy Game.

University	Collaboration With:	Collaborative Activities:
University of Groningen	Pharmacy assistants in training; Medical students	Consultations in the simulated pharmacy and pharmacy recruitment interviews; Simulates the role of general practitioners that consulted with the student-pharmacists to optimize patient treatments and medication utilization
Utrecht University	Teacher-pharmacists and general practitioners; Practicing pharmacists	Jointly training in interprofessional collaboration; Involved either as a staff member or simulated patients
Vrije Universiteit Brussel	Practicing pharmacists; Practicing physicians, midwifes, and nurses	Involved as a simulated patients; Contacting the pharmacy students regarding medication-related issues
University of Nottingham	Training pharmacists; Business school postgraduates; Dietetic students	Involved as a simulated patients; Assess and provide feedback on pharmacy business plans; Collaborate on a complex patient case
Griffith University	Practicing pharmacists; Academic staff from the School of Medicine	Involved as a simulated patients; Involved in case delivery, debriefing, and feedback
University of Bath	Practicing pharmacists	Involved as simulated patients
Vilnius University	General-practitioner-lecturer; Medical students	Assessor of clinical pharmacist’s cases; Involved as simulated patients

**Table 3:** Patient case (eczema), student outcomes.

Student Outcomes	University of Groningen	Utrecht University	University of Nottingham	Griffith University	University of Bath	Vilnius University
Best reported aspects	Patient questioning, history-taking	Patient-focused communication, explanation of not being able to provide a prescription medicine	Patient-focused communication, good dermatology and product knowledge	Patient-focused communication, including active listening and rapport-building	Detailed counselling on use of emollients and topical corticosteroids, including the application of finger tip units	Assessment of the condition, provided appropriate treatment and involved their patient in the treatment decision
Aspects for improvement	Non-pharmacological advice, the specific dosing information, conversation length	Trigger identification	Shared decision-making and non-pharmacological management advice	Detailed medication counselling, including provision of finger tip units advice with corticosteroid counselling	Differential diagnosis, shared decision-making and non-pharmacological management advice	Trigger identification, active listening and making the patient feel at ease
Appropriate (differential) diagnosis	71% (5 out of 7 pharmacies)	43% * (3 out of 7 pharmacies)	95% (19 out of 20 pharmacies)	87% (7 out of 8 pharmacies)	71% (12 out of 17 pharmacies)	100% (4 out of 4 pharmacies)

* The case was conducted at the begining of the game.

## Supplementary Materials

The following are available online at https://www.mdpi.com/article/10.3390/pharmacy9030125/s1, Table S1. Questionnaire—The Pharmacy Game at your university; Table S2. Simulated patient briefing for the eczema case; Supplementary File: Patient Case OTC Scenario-Eczema; Supplementary File: Ethical Clearance Statement; Table S3. Learning Outcomes of the Pharmacy Game participant universities; Table S4. The Pharmacy Game scoring across the participant universities; and Table S5. Potential therapeutic options and case assessment variables.

## Appendix A

**Table A1:** Free text comments of participants’ experiences with the Pharmacy Game.

University	Additional Participant Experiences
University of Groningen	At the beginning of the game, students are thrown into a deep waters. As the game progresses, they learn to swim, realize their strengthens and identify areas and competences for improvement. The nice thing about this educational concept is the fact that it allows implementation of actual and current situations. Therefore, it remains interesting for both the students and the lecturers, but in the same time challenging and labor intensive.
Utrecht University	Since 2016, there is a new curriculum Master Pharmacy. The teaching method ‘pharmacy game’ is now part of a 1st year course, instead of the 3rd year master course. Consequently, the learning goals as well as the course were adjusted accordingly. This questionnaire has been completed accounting for the “old” and “new” situation (2004–2016 and after 2016).
University of Nottingham	The game itself has undergone several iterations with the lastest being more focussed towards communication skills, teamwork, personal development, professional evolvement, leadership skills, coaching and feedback.
Griffith University	It has been a great experience for us but lots of work.
University of Bath	Once set up, a fantastic tool to help develop the students further in as real-life simulation as is possible! The students have commented how they found the experience enjoyable.
Vilnius University	We’ve played it only once yet, but from this game we’ve learned the following: students loved it, the game mirrors everyday pharmacy practice and gives possibility to use knowledge and skills in practice. The area that could be improved in pharmacy curriculum at VU-development of clinical communication skills.

## References

[1] Graafland M., Schraagen J.M., Schijven M.P. (2012). Systematic review of serious games for medical education and surgical skills training. BJS.

[2] Cain J., Piascik P. (2015). Are Serious Games a Good Strategy for Pharmacy Education?. Am. J. Pharm. Educ..

[3] Drummond D., Hadchouel A., Tesnière A. (2017). Serious games for health: Three steps forwards. Adv. Simul..

[4] Sauvé L., Renaud L., Kaufman D., Marquis J.-S. (2007). Distinguishing between Games and Simulations: A Systematic Review. J. Educ. Technol. Soc..

[5] Fens T., Dantuma-Wering C.M., Taxis K. (2020). The Pharmacy Game-GIMMICS^®^ a Simulation Game for Competency-Based Education. Pharm..

[6] Bedwell W.L., Pavlas D., Heyne K., Lazzara E.H., Salas E. (2012). Toward a Taxonomy Linking Game Attributes to Learning. Simul. Gaming.

[7] Okuda Y., Bryson E.O., DeMaria S., Jacobson L., Quinones J., Shen B., Levine A.I. (2009). The Utility of Simulation in Medical Education: What Is the Evidence?. Mt. Sinai J. Med. A J. Transl. Pers. Med..

[8] Gaba D.M., Calnan M.W., Sanford E. (2004). The future vision of simulation in health care. Qual. Saf. Health Care.

[9] Austin A., Rudolf F., Fernandez J., Ishimine P., Murray M., Suresh P., McDaniel M., Shishlov K., Oyama L. (2021). COVID-19 educational innovation: Hybrid in-person and virtual simulation for emergency medicine trainees. AEM Educ. Train..

[10] Kim B. (2015). Understanding Gamification.

[11] Dicheva D., Dichev C., Agre G., Angelova G. (2015). Gamification in Education: A Systematic Mapping Study. J. Educ. Technol. Soc..

[12] Hamari J., Koivisto J., Sarsa H. Does Gamification Work? A Literature Review of Empirical Studies on Gamification. Proceedings of the 47th Hawaii International Conference on System Sciences.

[13] Al-Azawi R., Al-Faliti F., Al-Blushi M. (2016). Educational Gamification Vs. Game Based Learning: Comparative Study. Int. J. Innov. Manag. Technol..

[14] van Der Werf J.J., Dekens-Konter J., Brouwers J.R. (2004). A New Model for Teaching Pharmaceutical Care Services Management. Pharm. Educ..

[15] Hirsch A.C., Parihar H.S. (2014). A Capstone Course with a Comprehensive and Integrated Review of the Pharmacy Curriculum and Student Assessment as a Preparation for Advanced Pharmacy Practice Experiences. Am. J. Pharm. Educ..

[16] Phillips B.B., Newsome A.S., Bland C., Palmer R., Smith K., DeRemer D.L., Phan S.V. (2019). Pharmacy Student Performance in a Capstone Course Utilizing the Pharmacists’ Patient Care Process. Am. J. Pharm. Educ..

[17] Harden R. (1999). What is a spiral curriculum?. Med. Teach..

[18] Deci E., Ryan R.M. (2008). Self-determination theory: A macrotheory of human motivation, development, and health. Can. Psychol..

[19] Orsini C., Evans P., Jerez O. (2015). How to encourage intrinsic motivation in the clinical teaching environment? A systematic review from the self-determination theory. J. Educ. Eval. Health Prof..

[20] Koster A.S., Schalekamp T., Meijerman I. (2017). Implementation of Competency-Based Pharmacy Education (CBPE). Pharmacy.

[21] Wolters M., van Paassen J., Minjon L., Hempenius M., Blokzijl M.-R., Blom L. (2021). Design of a Pharmacy Curriculum on Patient Centered Communication Skills. Pharmacy.

[22] Mylrea M.F., Gupta T.S., Glass B.D. (2017). Developing Professional Identity in Undergraduate Pharmacy Students: A Role for Self-Determination Theory. Pharmacy.

[23] Koster A.S., Mantel-Teeuwisse A.K., Woerdenbag H.J., Mulder W.M.C., Wilffert B., Schalekamp T., Buurma H., Wilting I., Westein M.P.D. (2020). Alignment of CanMEDS-based Undergraduate and Postgraduate Pharmacy Curricula in The Netherlands. Pharmacy.

[24] Bloom B.S., Engelhart M.D., Furst E.J., Hill W.H., Krathwohl D.R. (1956). Taxonomy of Educational Objectives, Handbook I: The Cognitive Domain.

[25] Anderson L.W., Krathwohl D.R. (2001). A Taxonomy for Learning, Teaching, and Assessing: A Revision of Bloom’s Taxonomy of Educational Objectives.

[26] Brookfield S. (1995). Becoming a Critically Reflective Teacher.

[27] Sillius A. (2005). GIMMICS: How to Organize, Manage and Control a Pharmacy Practice Game. E-Learning: Design, Development and Delivery.

[28] Van Rossem I., Devroey D., De Paepe K., Puttemans F., Petit P., Schol S., DeRidder S., Vandevoorde J. (2020). A Training Game for Students Considering Family Medicine: An Educational Project Report. J. Med. Life.

[29] Hope D., Rogers G., Grant G., King M. (2021). Experiential Learning in a Gamified Pharmacy Simulation: A Qualitative Exploration Guided by Semantic Analysis. Pharmacy.

[30] Schaafsma E., Dantuma C., Pilon K., de Gier H. GIMMICS: A simulation of pharmacy practice. Proceedings of the ESCP–SFPC International Workshop Acquisition of Pharmaceutical Skills: Simulation, Serious Games, Innovative Approaches.

[31] Schaafsma E., Dantuma-Wering C., Van Wieren D., Sarre S., De Paepe K. A Pharmacy Game: Experiencing the Possibilities of an Active and Reflective Learning Style. Proceedings of the 16th International Social Pharmacy Workshop: Communication and Information in Pharmacy.

[32] Heersche A., Hazen A.C.M., van Paassen J.G., Schalekamp T., Verdel B.M., van Wieren-de Wijer B.M.A., Bouvy M.L. GIMMICS: A Bridge between Academic Learning and Community Pharmacy. Proceedings of the European Association of Faculties of Pharmacy Annual Conference.

[33] Verdel B.M., van Wieren-de Wijer B.M.A., van Paassen J.G., Hazen A.C.M., Bouvy M.L. GIMMICS: A Pharmacy Game in an Academic Setting. Proceedings of the Monash Pharmacy Education Symposium: Teaching for Learning.

[34] Boyd M., Solanki V., Anderson C., Sonnex K., Brydges S. Pharmacy Leadership and Management Module: An Evaluation of the Student Experience and Its Perceived Usefulness for Future Employment. Proceedings of the Monash Pharmacy Education Symposium.

[35] Boyd M., Solanki V., Anderson C., Sonnex K., Brydges S. Pharmacy Leadership and Management: A New High Fidelity Simulation to Prepare Students for Their Future Practise. Proceedings of the Monash Pharmacy Education Symposium.

[36] Solanki V., Boyd M., Anderson C., Sonnex K., Brydges S. Using Performance and Leadership Mentors to Support Students during a Simulated Pharmacy Business Module. Proceedings of the Monash Pharmacy Education Symposium.

[37] Solanki V., Boyd M., Sonnex K., Brydges S., Anderson C. Pharmacy Leadership and Management: Student Perspectives of Team-Working in a Simulated Pharmacy Business Module. Proceedings of the Pharmacy Education Conference.

[38] Hope D.L., Rogers G.D., Grant G.D., King M.A. Affective Learning in a Serious Pharmacy Game. Proceedings of the European Association of Faculties of Pharmacy Conference.

[39] Hope D.L., Rogers G.D., Grant G.D., King M.A. Impact of a Serious Pharmacy Game on Senior Students’ Professional Competencies: A Controlled Trial. Proceedings of the European Association of Faculties of Pharmacy Conference.

[40] Hope D.L., Rogers G.D., Grant G.D., King M.A. Ecological Momentary Assessment in a Gamified Simulation. Proceedings of the European Association of Faculties of Pharmacy Conference.

[41] Hope D.L., Rogers G.D., Grant G.D., King M.A. Experiential Learning in a Gamified Simulation Detected by Semantic Analysis. Proceedings of the European Association of Faculties of Pharmacy Conference.

[42] Hope D., Rogers G., Grant G., King M. Can an Extended Immersive Pharmacy Simulation Game Influence Students’ Perceptions of Their Professional Competencies?. Proceedings of the Health Services Research and Pharmacy Practice Conference.

[43] Babar Z.-U.-D. (2015). Pharmacy Practice Research Methods.

[44] Ogrinc G., Armstrong G.E., Dolansky M.A., Singh M.K., Davies L. (2019). SQUIRE-EDU (Standards for QUality Improvement Reporting Excellence in Education). Acad. Med..

[45] Sandelowski M. (2000). Whatever Happened to Qualitative Description?. Res. Nurs. Health.

[46] Given L. (2008). The SAGE Encyclopedia of Qualitative Research Methods.

[47] Hsieh H.-F., Shannon S.E. (2005). Three approaches to qualitative content analysis. Qual. Health Res..

[48] European Commission European Credit Transfer and Accumulation System (ECTS). https://ec.europa.eu/education/resources-and-tools/european-credit-transfer-and-accumulation-system-ects_en.

[49] Miller G.E. (1990). The assessment of clinical skills/competence/performance. Acad. Med..

[50] Seybert A.L., Smithburger P.L., Benedict N.J., Kobulinsky L.R., Kane-Gill S.L., Coons J.C. (2019). Evidence for simulation in pharmacy education. J. Am. Coll. Clin. Pharm..

[51] Aburahma M.H., Mohamed H.M. (2015). Educational Games as a Teaching Tool in Pharmacy Curriculum. Am. J. Pharm. Educ..

[52] Patel J. (2008). Using Game Format in Small Group Classes for Pharmacotherapeutics Case Studies. Am. J. Pharm. Educ..

[53] Barclay S.M., Jeffres M.N., Bhakta R. (2011). Educational Card Games to Teach Pharmacotherapeutics in an Advanced Pharmacy Practice Experience. Am. J. Pharm. Educ..

[54] Tietze K.J. (2007). A Bingo Game Motivates Students to Interact with Course Material. Am. J. Pharm. Educ..

[55] Chen A.M.H., Plake K.S., Yehle K.S., Kiersma M.E. (2011). Impact of the Geriatric Medication Game on Pharmacy Students’ Attitudes Toward Older Adults. Am. J. Pharm. Educ..

[56] Roche V.F., Alsharif N.Z., Ogunbadeniyi A.M. (2004). Reinforcing the Relevance of Chemistry to the Practice of Pharmacy through the Who Wants To Be A Med Chem Millionaire?. Learning Game. Am. J. Pharm. Educ..

[57] Oliver C.H., Hurd P.D., Beavers M., Gibbs E., Goeckner B., Miller K. (1995). Experiential Learning About the Elderly: The Geriatric Medication Game. Am. J. Pharm. Educ..

[58] Evans S., Lombardo M., Belgeri M., Fontane P. (2005). The Geriatric Medication Game in Pharmacy Education. Am. J. Pharm. Educ..

[59] Neill M.A., Wotton K. (2011). High-Fidelity Simulation Debriefing in Nursing Education: A Literature Review. Clin. Simul. Nurs..

[60] Aura S.M., Sormunen M., Jordan S.E., Tossavainen K.A., Turunen H. (2015). Learning Outcomes Associated with Patient Simulation Method in Pharmacotherapy Education. Simul. Health J. Soc. Simul. Health.

[61] Kapralos B., Johnston C., Finney K., Dubrowski A. (2011). A Serious Game for Training Health Care Providers in Interprofessional Care of Critically-Ill and Chronic Care Patients. J. Emerg. Technol. Web Intell..

[62] Schindel T.J., Yuksel N., Breault R., Daniels J., Varnhagen S., Hughes C.A. (2017). Perceptions of pharmacists’ roles in the era of expanding scopes of practice. Res. Soc. Adm. Pharm..

[63] Australian Pharmacy Council Accreditation Standards for Pharmacy Programs. https://www.pharmacycouncil.org.au/resources/pharmacy-program-standards/.

[64] World Health Professions Alliance Interprofessional Collaborative Practice. https://www.whpa.org/activities/interprofessional-collaborative-practice.

[65] Vuurberg G., Vos J., Christoph L., de Vos R. (2019). The effectiveness of interprofessional classroom-based education in medical curricula: A systematic review. J. Interprofessional Educ. Pract..

[66] Guraya S.Y., Barr H. (2018). The effectiveness of interprofessional education in healthcare: A systematic review and meta-analysis. Kaohsiung J. Med. Sci..

[67] Vyas D., Bray B.S., Wilson M.N. (2013). Use of Simulation-based Teaching Methodologies in US Colleges and Schools of Pharmacy. Am. J. Pharm. Educ..

[68] Masters C., Baker V.O., Jodon H. (2013). Multidisciplinary, Team-Based Learning: The Simulated Interdisciplinary to Multidisciplinary Progressive-Level Education (SIMPLE©) Approach. Clin. Simul. Nurs..

[69] Ponte P.R., Gross A.H., Milliman-Richard Y.J., Lacey K. (2010). Interdisciplinary Teamwork and Collaboration an Essential Element of a Positive Practice Environment. Annu. Rev. Nurs. Res..

[70] Hedges A.R., Johnson H.J., Kobulinsky L.R., Estock J.L., Eibling D., Seybert A.L. (2019). Effects of Cross-Training on Medical Teams’ Teamwork and Collaboration: Use of Simulation. Pharmacy.

[71] Cropp C.D., Beall J., Buckner E., Wallis F., Barron A. (2018). Interprofessional Pharmacokinetics Simulation: Pharmacy and Nursing Students’ Perceptions. Pharmacy.

[72] Abraham O., Feathers A., Grieve L., Babichenko D. (2019). Developing and piloting a serious game to educate children about over-the-counter medication safety. J. Pharm. Health Serv. Res..

[73] Dankbaar M.E.W., Richters O., Kalkman C.J., Prins G., Cate O.T.J.T., Van Merrienboer J.J.G., Schuit S.C.E. (2017). Comparative effectiveness of a serious game and an e-module to support patient safety knowledge and awareness. BMC Med. Educ..

[74] Graudins L.V., Dooley M.J. (2016). Medication Safety: Experiential Learning for Pharmacy Students and Staff in a Hospital Setting. Pharmacy.

[75] Richey Smith C.E., Ryder P., Bilodeau A., Schultz M. (2016). Use of an Online Game to Evaluate Health Professions Students’ Attitudes toward People in Poverty. Am. J. Pharm. Educ..

