# Supplementary material for: The International Pharmacy Game: A Comparison of Implementation in Seven Universities World-Wide
Source: Pharmacy (Basel). 2021 Jul 13;9(3):125. doi: 10.3390/pharmacy9030125 (PMC8293378; doi:10.3390/pharmacy9030125)
Supplement: Supplementary file 1 [file pharmacy-09-00125-s001.zip › pharmacy-1186770-supplementary.pdf]

pharmacy

MDPI

Supplementary files:

Table S1. Questionnaire - The Pharmacy Game at your university

Questionnaire details
**Contact information:**<br>1. Primary contact person for the Pharmacy Game (name and surname):<br>2. University:<br>3. e-mail:<br>4. Other persons involved and their contact e-mails:<br>5. Since when are you using the Pharmacy Game (year)?<br>6. Which name did you give to your Pharmacy Game?
**General information about utilization of your Pharmacy Game:**<br>7. Do you use CAMS (case management system)? (select one or more options)<br>a) Yes<br>b) No time to include this yet<br>c) We have an alternative way to manage this part of the game<br>d) Other (please specify)<br>8. Do you use ARS (actor registration system)? (select one or more options)<br>a) Yes<br>b) No time to include this yet<br>c) We have an alternative way to manage this part of the game<br>d) Other (please specify)<br>9. Do you use the prescription generator? (select one or more options)<br>a) Yes<br>b) No time to include this yet<br>c) We have an alternative way to manage this part of the game<br>d) Other (please specify)<br>10. Are you using your Pharmacy Game for training of: (select one or more options)?<br>a) Pharmacy students<br>b) Medical students<br>c) Other (please specify)<br>11. Is your Pharmacy Game a mandatory course for your students? YES NO<br>12. Are you using your Pharmacy Game as: (select one or more options)?<br>a) standalone course<br>b) integrated in a larger course<br>c) other (please specify)<br>13. Are there ECTS [European Credit Transfer and Accumulation System] associated with your Pharmacy Game?<br>a) No<br>b) Yes (please specify how many ECTS)
**Gaming frequencies:**<br>14. How many times do you organize your Pharmacy Game in one academic year (number of games per year)?<br>15. What is the duration of your Pharmacy Game in days?<br>16. How many students are involved per game?<br>17. How many students manage one pharmacy?<br>18. Please provide the number of full time equivalent (FTE) teaching staff members (e.g., lecturers) who are involved in teaching one game.<br>19. What is the background of the teaching staff who are involved in your Pharmacy Game?<br>a) Pharmacist<br>b) Pharmacist assistant/technician<br>c) Physician<br>d) Other (please specify)

20. Which other staff (e.g. IT services, secretary, student assistants) is involved in your Pharmacy Game? Please provide the number and their roles
21. Do you have externals included in your Pharmacy Game (e.g. actors, students, patients)?
    a) No
    b) Yes (please specify)

Learning outcomes:

22. What are the main learning goals you want to achieve using the Pharmacy Game in your educational program?
23. With the following questions we would like to know more about the methods that you use to assess the performance of your students in groups (each pharmacy) and individually:
24. Do you use methods to assess a student's performance as a group (each pharmacy)?
    a) Yes *
    b) No

    If yes, please list the methods and explain how they are used.
25. Do you use methods to assess a student's individual performance?
    a) Yes *
    b) No

    *If yes, please list the methods and explain how they are used.

General questions:

Would you like to share with us any additional experiences with the Pharmacy Game?

This questionnaire was completed by:

Table S2. Simulated patient briefing for the patient eczema case

The simulated patient/actor shall give the following information when initially asked	The simulated patient/actor shall give the following information when specifically asked	If asked for any other questions
• he/she needs advice about its child's skin<br>• he/she thinks that it seems to be causing it some irritation and have noticed it scratching quite a bit recently<br>• he/she don't know what has caused it	• they have a patch on the inside of child's elbow<br>• the skin looks dry and has gone darker than it usually is in places<br>• it started as just a small patch, but it has spread over the last week<br>• the child is 11 years old<br>• the child does not take any medicines or have any medical conditions<br>• he/she hasn't tried anything on it so far	• ask if it is likely to come back again

Supplementary file: Patient Case OTC Scenario - Eczema

Background

For this scenario, you are required to role play as a patient and visit simulated pharmacies for advice on the same problem.

You will be required to mark the student on how they dealt with the task and how they made you feel as a patient.

Important information:

- The patient should be based on yourself for gender and age. You may change other personal details and behave how you chose (polite, happy, thankful, frustrated, angry, short tempered etc…) but please remain consistent for all pharmacies.
- Information should only be given to the student if an open question is asked or if they ask specifically about a particular aspect.
- You will contact the pharmacies using Microsoft Teams Video Call.
    - A 10-minute meeting will have been set up for each pharmacy for you by the PA40330 Unit Staff.
- Scenarios are designed to take no longer than 10 minutes and you should aim to keep to this time.
    - If students are taking too long, then please leave the call!

Patient Brief

Call the pharmacy and ask if you can ask for the pharmacist's advice.

Only give the following information if asked initially:

- You would like some advice about your child's skin

- It seems to be causing them some irritation and you have noticed them scratching quite a bit recently
- You don't know what has caused it

Only give the following information if asked specifically:

- They have a patch on the inside of their elbow
- The skin looks dry and has gone darker than it usually is in places
- It started as just a small patch but it has spread over the last week
- Your child is 11 years old
- They don't take any medicines or have any medical conditions
- You haven't tried anything on it so far

You have a picture of the patch to show if asked.

If asked if you have any questions:

- Is it likely to come back again?

<u>**UK Options for Treatment**</u>

- Frequent and liberal use of an emollient (e.g. E45 cream, Cetraben cream) should be encouraged
- Try to identify if there are any trigger factors such as certain clothing, soaps or detergents, animals and heat
- As child is over 10 years old, hydrocortisone 1% cream could be sold to help with the flare
    - Treatment should be used for a maximum of 1 week
    - Should continue treatment for 48 hours after the flare has been controlled
- Should try to avoid scratching the area and simply rub the area with the fingers to alleviate itch

Picture:

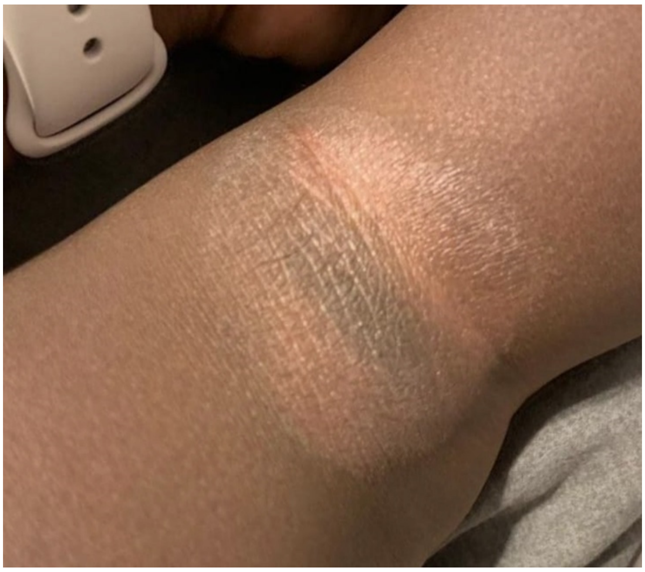

Supplementary file: Ethical clearance statement

This research was conducted in compliance with all relevant national and international laws, rules and agreements. The research does not falls under the the Medical Research Involving Human Participants Act (WMO) or any other applicable laws and regulations that impose conditions on the research, such as the Regulation on the protection of natural persons with regard to the processing of personal data and on the free movement of such data, and repealing Directive 95/46/EC; General Data Protection Regulation (GDPR/AVG) and the Dutch Implementation Act "Uitvoeringswet Algemene verordening gegevensbescherming" (UAVG, as the study did not directly concern humans or animals, and did not offend the privacy rights of any concerned party).

The research team of the University of Groningen, that initially launched the study is trained to practice scientific integrity and lead this study in line with the university codes of conduct:
- Netherlands Code of Conduct for Research Integrity
- University of Groningen (UG) Academic Integrity Code 2020
- Regulations for the Protection of Academic Integrity UG

Each participant of the study provided informed verbal or written consent during a regular meeting of the consortium. Each university appointed a key person that would contribute to this research by completing the questionnaire. Distribution of the questionnaire was accompanied with a research statement describing the purpose of the study and how the data would be used. When the questionnaire was prepared, it was shared only with the contacts that had already agreed to participate. Moreover, each of the participants is listed as a co-author or collaborator (in acknowledgments) to this research study.

Table S3. Learning Outcomes of the Pharmacy Game participant universities

University	Learning Outcomes for the Pharmacy Game
University of Groningen	• Collaboration*- within the pharmacy team and with other healthcare providers<br>• Leadership and organization-* leading from a vision, taking responsibility, entrepreneurship and innovation, coaching and managing individuals<br>• Communication-* with patients, with other health care providers and with student's pharmacy team, both verbally and in writing<br>• Pharmaceutical expertise*- being able to reflect, process prescriptions, apply guidelines, show clinical thinking; make well-considered pharmacotherapeutic choices based on clinical reasoning and substantiating - using medical jargon - especially when treatment according to the guidelines is not possible
Utrecht University (curriculum before September 2016)	• To develop management skills (strategic, financial, logistic, leadership)<br>• To be aware of business and legal aspects necessary for pharmaceutical patient care and medication policy<br>• Communication skills: to function as a (pharmacy) team, to communicate effectively with patients and healthcare professionals both verbally and written<br>• To optimize pharmacotherapeutic care in collaboration with prescribers in terms of quality, effectiveness, and efficiency
Utrecht University (curriculum after September 2016)	• The course bridges the gap between the knowledge and insights gained in the first three master courses and the first internship in the community pharmacy<br>• To gain insight in the complexity of the task areas of a pharmacist<br>• Learning goals: fully process a prescription and adequately implement pharmaceutical patient care based on clinical situation and current guidelines and evidence; to conduct consultations in a patient-oriented manner<br>• Gain experience in: communication with healthcare providers an patients; functioning as a team and working with others on the basis of trust and respect; the structure and financing of the health care system; laws and regulations
Vrije Universiteit Brussel	• As students do their internships on different places, the pharmacy game is a perfect way for them to speak to each other and become aware of different workflows. As such we can also eliminate differences between the students.<br>• Additionally, we select a number of cases that are rather rare of which we know they definitely don't come into contact within each pharmacy. This way we know they all got into contact with these specific cases.<br>o The student learned to test his own insights against those of others and is able to evaluate and describe his own functioning within a group. The student is able to provide structural feedback to fellow students.<br>o The student is able to reflect on his / her own performance within a professional team and communicate about this in writing. The student is also able to process and use it constructively.
University of Nottingham	• To bring together learning from across the whole Master of Pharmacy course.<br>• Allow students to capitalise on experiences gained outside the module such as vocational experiences both within the course and outside.<br>• To further develop leadership, management, marketing, communication skills, problem-solving abilities and working in teams.<br>• Design and develop learning and development resources for year 1-2 students via organised placements within their pharmacies.<br>• Contribute to the education and training of other members of the team, including peer review and assessment (SHOWS HOW)**<br>• Contribute to the development of other members of the team through coaching and feedback (KNOWS HOW)**<br>• To run a simulated business that provides patient care, essential, advanced, and bespoke services.
Griffith University	• Evaluate advanced practice-based cases to apply legal, ethical and clinical reasoning. (SHOWS HOW)**<br>• Apply tailored communication skills required for all pharmacy practice related consultations, to deliver person-centred care. (SHOWS HOW)**

	• Demonstrate core drug literacy by explaining the drug class, indications and key counselling points of the frequently-prescribed medicines considered relevant to contemporary pharmacy practice. (SHOWS HOW)*<br>• Integrate knowledge for safe and appropriate delivery of primary and preventative healthcare for special population patients addressed in this course. (KNOWS)**<br>• Evaluate complex practice-based cases to dispense medicines, including assessment of the legal, Pharmaceutical Benefits Scheme (PBS) and clinical appropriateness of the prescribed medicines, applying a systematic dispensing procedure and counselling of patients/ carers. (SHOWS HOW)**<br>• Apply research evidence to deliver pharmaceutical health education and training. (SHOWS HOW)**<br>• Collaborate in teams to plan, manage and conduct simulated pharmacies to deliver holistic person-centred care. (DOES)**<br>• Demonstrate understanding of work readiness concepts as they apply to contemporary pharmacy practice. (KNOWS HOW)**
University of Bath	• Apply professional judgement in the best interests of the patient and the public.<br>• Undertake effective patient centred consultations.<br>• Actively support patients and their carers in the safe and effective use of their medicines and devices.<br>• Manage and utilise resources in order to ensure efficient work flow and minimise risk in the workplace.<br>• Demonstrate effective negotiation skills.<br>• Communicate and work effectively with other members of a pharmacy team.<br>• Demonstrate good organisational skills to complete tasks to a professional standard.<br>• Respond effectively to complaints, incidents and errors and in a manner which demonstrates patient-centred care.<br>• Recognise conflicts of interest and utilises appropriate standards of practice.<br>• Demonstrate an ability to manage resources effectively and participate in financial planning processes, including the production of a business plan.<br>• Respond with flexibility and adaptability to new situations and change.<br>• Reflect on and recognise own limitations, work safely and seek support where appropriate.
Vilnius University	• To bring together learning from across the whole Master of Pharmacy course.<br>• Gain pharmacy practice skills.<br>• Gain oral and written communications skills.<br>• Learn to work as a team member towards common goal.<br>• Practice pharmacy management skills.<br>• Develop competency development plan.

* The learning outcomes are based on the Pharmacist Competency Framework & Domain-specific Frame of Reference for the Netherlands [35]

** Bracketed verbs following each learning objective reflect the expected level of application, from Miller's (1990) pyramid of clinical assessment [34].

Table S4. The Pharmacy Game scoring across the participant universities

Score*	University of Groningen	Utrecht University before/after 2016	Vrije Universiteit Brussel**	University of Nottingham	Griffith University	University of Bath	Vilnius University
**Starting Score**	8,000	10,000	🟢🟡🔴	8,000	10,000	10,000	8,000
**Team norms contract**	-	-	-	-	⩽1,000	-	-
**Team business plan**	+/-100	+/- 150 / N.A.	-	+/-100	-	20%[g]	-
**Team annual report**	+/-100	+/- 75 / N.A.	-	+/-100	-	-	-
**Pharmacy opening**	+/-40	+/- 30 / N.A.	-	-	-	+/-50	-
**Prescriber communication**	+/-20[i]	+/- 20	🟢🟡🔴	+/-20	+/-100	-	+/-30
**Patient case – face to face**	+/-40[i]	+/- 30	🟢🟡🔴	0-40	+/-200	+/-50	+/-60
**Patient case – telephone**	+/-40[i]	+/- 30	🟢🟡🔴	0-40	+/-100	+/-50	+/-60
**Patient case – email/post**	+/-40[i]	+/- 30	-	0-40	-	-	+/-60
**Veterinary case**	-	-	🟢🟡🔴	0-40	-	-	-
**Compounding – general**	-	-	🟢🟡🔴	-	-	-	-
**Compounding – challenging**	-	-	Y	-	-	-	-
**Vacinations case**	-	-	-	0-40	-	-	-
**Prescriptions**	+/-60	+/- 250	🟢🟡🔴	+/-100	+/-100	+/-200	+/-10[x]
**Audio/video recorded patient**	-	-	🟢🟡🔴	0-40	+/-200	-	+/-60
**Pharmacy ordering** (Case	+/-40[i]	-	-	0-40	-	-	-
**Out of hours service case**	+/-40[i]	+/- 30	🟢🟡🔴	0-40	-	-	+/-100
**Medication review meeting 1 –**	+/-20[i]	-	🟢🟡🔴	-	-	-	-
**Medication review meeting 2 –**	+/-40[i]	-	🟢🟡🔴	-	-	-	-
**Medication review – written**	-	+/-	🟢🟡🔴	0-40	⩽300	+/-50	-
**Quality – risk analyses**	+/-40	+/-	-	-	-	-	-
**Quality – pharmacy audit**	+/-40	-	-	-	-	-	-
**Quality – use of quality**	+/-1	-	-	-	-	-	-
**Quality – complaints and errors**	+/-40	+/- 50	-	0-40	-	+/-50	+/-30
**Quality – daily barcode**	-	-	🟢🟡🔴	-	⩽100	-	-
**Return of medications**	-	-		0-40	-	-	-
**Disposal of medications**	-	-		0-20	-	-	-
**Controlled drug register**	-	-	-	0-40	⩽200	+/-50	-
**Adverse drug reaction written**	-	-	🟢🟡🔴	-	⩽500	10%[g]	-
**Euthanasia case requested**	+/-20[i]	+/-	🟢🟡🔴	-	-	-	-
**Euthanasia case proceed**	+/-40[i]	+/-	🟢🟡🔴	-	-	-	-
**Staff recruitment – position**	+/-40	+/-	-	0-20	-	-	-
**Staff recruitment – job**	+/-60	+/- 50	-	0-20	-	-	-
**Appointments with pharmacy**	+/-40	-	🟢🟡🔴	-	-	-	-
**Post academic education**	+/-40	-	Y	-	-	-	-
**FTO preparation**	+/-20	-	-	-	-	-	-
**FTO content**	+/-40	-	-	-	-	-	-
**FTO presentation/execution**	+/-40	+/- 100/ N.A.	-	-	-	-	-
**FTO minutes**	+/-20	-	-	-	-	-	-
**Innovation project**	+100	+/- 100/ N.A.	Pitch	+100	-	-	+/-100
**Small project**	+/-40	+/- 50	Podcast&SM	0-40	+/-100	+/-50	+/-40
**Big project**	+/-100	+/- 100	Infographic	0-100	+/-500	+/-100	+/-100
**Factcheck of a newspaper**	-		Article	-			-
**Individual reflective journals**	-	-	Y	-	⩽500	20%[g]	-

**Team debrief and reflection**	-	N.A./max.75	Y	-	⩽500	-	-
**Extended research CPD**	-	-	-	-	⩽10,000	-	-
**Tablet/capsule identification**	-	+/- 75/ N.A.	-	-	+/-50	-	-
**Bonus/penalty**	-	-	-	-	+/-50	+/-up to 100	+/-100
**Other *ad hoc* activities**	-	+/- 50/ N.A.	-	0-40	+/-50	+/-50	+/-100
**Mean winning score**	10,689	-	-	9,120	52,190	11,600	14,000

* all scores are reflected as number of patients earned or lost
**Vrije Universiteit Brussel use a traffic light system for each case, where green= as it should be, orange=incomplete but no harm is done, red= mistakes are made with possible harm to the patient

g=not marked within the game itself but it is graded as a piece of coursework (% of overall unit mark)
i = cases scored by individual student
x=scored per prescription

Y- students do the activity and therefore receive written feedback
FTO = pharmacotherapeutic consultation with general practitioner
CPD = continuing professional development
N.A.= not applicable
SM=Social Media

Table S5. Potential therapeutic options and case assessment variables

	University of Groningen	Utrecht University	Vrije Universiteit Brussel**	University of Nottingham	Griffith University	University of Bath	Vilnius University
**Potential Therapeutic Options**							
Pharmacological Management							
Recommend OTC mild-moderate topical corticosteroid* e.g. hydrocortisone, clobetasone	N	N	Y	Y	Y	Y	Y
Recommend OTC moisturiser e.g. cetomacrogol, sorbolene, white soft paraffin	Y	Y	Y	Y	Y	Y	Y
Refer for Rx for mild-moderate-potent topical corticosteroid e.g. hydrocortisone, clobetasone, mometasone, methylprednisolone	Y	Y	Y	Y	Y	Y	Y
Non-pharmacological Management							
Avoid triggers	Y	Y	Y	Y	Y	Y	Y
Avoid scratching, heat etc	Y	Y	Y	Y	Y	Y	Y
Use soap-free alternatives	Y	Y	Y	Y	Y	Y	Y
**Case Assessment Variables**							
Questions and history taking	Y	Y	Y	Y	Y	Y	Y
Differential diagnosis				Y	Y	Y	Y
Appropriate pharmacological product	Y	Y	Y	Y	Y	Y	Y
Dose information	Y	Y	Y	Y	Y	Y	Y
Potential adverse events	Y	Y	Y	Y	Y	Y	Y
Appropriate non-pharmacological advice	Y	Y	Y	Y	Y	Y	Y
Communication	Y	Y	Y	Y	Y	Y	Y
Manage patient question/s	N	N	Y	N	Y	N	Y
Appropriate medical referral	N	N	Y	Y	Y	Y	N

OTC = over the counter; Rx = prescription; N= no, Y = Yes
